# Immune profiling of patients with extranodal natural killer/T cell lymphoma treated with daratumumab

**DOI:** 10.1007/s00277-023-05603-w

**Published:** 2024-01-18

**Authors:** Min Qing, Tianyuan Zhou, Tatiana Perova, Yann Abraham, Cheryl Sweeney, Maria Krevvata, Xiaokang Zhang, Ming Qi, Grace Gao, Tae Min Kim, Ming Yao, Seok-Goo Cho, Hyeon Seok Eom, Soon Thye Lim, Su-peng Yeh, Yok Lam Kwong, Dok Hyun Yoon, Jin Seok Kim, Won Seog Kim, Longen Zhou, Ricardo Attar, Raluca I. Verona

**Affiliations:** 1 Janssen Research & Development, Shanghai, China; 2 Janssen Research & Development, LLC, Spring House, PA USA; 3 Janssen Research & Development, LLC, Beerse, Belgium; 4 Johnson & Johnson (Ireland), Dublin, Ireland; 5 Janssen Research & Development, Beijing, China; 6 Seoul National University Hospital, Seoul, South Korea; 7 National Taiwan University Hospital, Taipei, Taiwan; 8 Seoul St. Mary’s Hospital, College of Medicine, The Catholic University of Korea, Seoul, South Korea; 9 National Cancer Center, Goyang-si, South Korea; 10 Division of Medical Oncology, National Cancer Centre Singapore, Singapore, Singapore; 11 China Medical University Hospital, Taichung, Taiwan; 12 Queen Mary Hospital, Pok Fu Lam, Hong Kong; 13 Asan Medical Center, University of Ulsan College of Medicine, Seoul, South Korea; 14 Yonsei University College of Medicine, Severance Hospital, Seoul, South Korea; 15 Division of Hematology/Oncology, Department of Medicine, Sungkyunkwan University School of Medicine, Samsung Medical Center, Seoul, South Korea

**Keywords:** Natural killer/T cell lymphoma, Daratumumab, Biomarkers, Immune profiling

## Abstract

Natural killer/T cell lymphoma (NKTCL) is a highly aggressive, heterogeneous non-Hodgkin lymphoma resulting from malignant proliferation of cytotoxic natural killer (NK) or T cells. Previous studies demonstrated variable expression of CD38 on NKTCL tumors. Daratumumab, a human IgGκ monoclonal antibody targeting CD38 with a direct on-tumor and immunomodulatory mechanism of action, was hypothesized to be a novel therapeutic option for patients with relapsed or refractory (R/R) NKTCL. In the phase 2 NKT2001 study (ClinicalTrials.gov Identifier: NCT02927925) assessing the safety and efficacy of daratumumab, a suboptimal overall response rate was seen in R/R NKTCL patients. One patient, whose tumors did not express CD38, responded to treatment, suggesting that the immunomodulatory activities of daratumumab may be sufficient to confer clinical benefit. To understand the suboptimal response rate and short duration of response, we investigated the immune profile of NKTCL patients from NKT2001 in the context of daratumumab anti-tumor activity. Tumor tissue and whole blood were, respectively, analyzed for CD38 expression and patient immune landscapes, which were assessed via cytometry by time-of-flight (CyTOF), multiparameter flow cytometry (MPFC), clonal sequencing, and plasma Epstein-Barr virus (EBV)-DNA level measurements. Changes observed in the immune profiles of NKTCL patients from NKT2001, including differences in B and T cell populations between responders and nonresponders, suggest that modulation of the immune environment is crucial for daratumumab anti-tumor activities in NKTCL. In conclusion, these findings highlight that the clinical benefit of daratumumab in NKTCL may be enriched by B/T cell–related biomarkers.

**Supplementary Information:**

The online version contains supplementary material available at 10.1007/s00277-023-05603-w.

## Introduction

Natural killer/T cell lymphoma (NKTCL) is an aggressive, heterogeneous type of non-Hodgkin lymphoma characterized by malignant proliferation of cytotoxic natural killer (NK) or T cells [1, 2]. The malignancy presents primarily in extranodal sites and is associated with Epstein-Barr virus (EBV) infection [2, 3]. For patients with stage I/II NKTCL, the standard of care is an asparaginase-containing regimen combined with radiotherapy, with sequential chemotherapy and radiotherapy most commonly employed. For patients with stage III/IV NKTCL, the standard treatment regimen contains dexamethasone, methotrexate, ifosfamide, L-asparaginase, and etoposide (SMILE) [4]. While asparaginase-containing chemotherapeutic regimens have improved outcomes for patients with NKTCL, patients with relapsed/refractory (R/R) NKTCL typically do not respond to standard chemotherapy-based regimens. With limited treatment options, the prognosis of patients with R/R NKTCL is poor, with a reported median overall survival of approximately 6 months [4]. Thus, a great unmet medical need exists for this patient population.

Daratumumab, a human IgG_κ_ monoclonal antibody targeting CD38 with a direct on-tumor [5–8] and immunomodulatory [9–11] mechanism of action, demonstrates greater cytotoxicity of multiple myeloma (MM) cells ex vivo compared with analogs of other CD38 antibodies [12], and is approved for MM treatment in many countries worldwide [13, 14]. Previous studies have demonstrated variable expression of CD38 on NKTCL tumors, with high expression observed in approximately 50% of tumor samples [15]. Therefore, daratumumab was hypothesized to be a novel therapeutic option for patients with R/R NKTCL.

The open-label, single-arm, multicenter, phase 2, NKT2001 study (ClinicalTrials.gov Identifier NCT02927925) assessed the safety and efficacy of daratumumab in Asian patients with R/R extranodal NKTCL, nasal type [16]. Patients achieved an overall response rate (ORR) of 25.0%, with a median duration of response of 55.0 days, suggesting that daratumumab monotherapy is insufficient to treat R/R NKTCL with aggressive features [16]. Based on the totality of data, the NKT2001 study did not proceed with expansion because the response rate did not meet the prespecified target of 30% ORR and lacked durable responses. However, we found that baseline B cell numbers in responders to daratumumab were higher than in nonresponders, while baseline plasma EBV-DNA, tumor CD38 expression, and NK cell counts did not show a clear correlation with clinical response [16]. Of note, 1 patient whose tumors did not express CD38 responded to daratumumab, suggesting the immunomodulatory activities of daratumumab may confer clinical benefit [17]. Here, we investigate the immune profiles of patients with R/R extranodal NKTCL, nasal type, from the NKT2001 study, in the context of daratumumab anti-tumor activity to understand the suboptimal response rate and short response duration.

## Methods

### Sample sources

The complete methodology of the NKT2001 study was previously reported [16]. Briefly, patients were enrolled from 14 clinical study sites across 5 countries/regions including Korea, China, Singapore, Taiwan, and Hong Kong Special Administrative Region. Eligible patients had histologically confirmed extranodal NKTCL, nasal type, classified according to the World Health Organization classification [18], were refractory to or relapsed after achieving complete or partial remission on ≥ 1 line of chemotherapy, and were not candidates for other treatment modalities. Other key eligibility criteria included ≥ 1 measurable disease site [assessed by positron emission tomography (PET) scan for positive uptake of ^18^F-fluorodeoxyglucose (FDG) in nodal or extranodal sites]; an Eastern Cooperative Oncology Group performance status (ECOG PS) score of 0 to 2; and a life expectancy of ≥ 3 months.

Patients received 28-day cycles of intravenous daratumumab 16 mg/kg once weekly during cycles 1 and 2, every 2 weeks during cycles 3 through 6, and every 4 weeks thereafter until disease progression, unacceptable toxicity, or patient withdrawal. Disease evaluations via radiological [computed tomography (CT) or magnetic resonance imaging] and PET-CT (whole-body ^18^F-FDG PET-CT) scans occurred at screening, every 8 weeks (± 7 days) for the first 6 months, and every 16 weeks (± 7 days) thereafter until disease progression, withdrawal, or end of study. Patients were classified as responders (defined as patients with best clinical response of partial response or better) or nonresponders. Blinded independent central review was performed to review imaging data and clinical information per a predefined independent central review charter. Central reviewers assessed disease status based on the Revised Criteria for Response Assessment: Lugano classification [19].

A total of 32 patients were enrolled (7 patients from mainland China; 25 patients enrolled outside of mainland China). The effect of daratumumab on ORR was evaluated using Simon’s two-stage design [16]. Clinical response and biomarker data were analyzed from patients who were enrolled outside of mainland China and received daratumumab with the clinical cutoff date of 9 October 2019. Baseline CD38 expression levels on tumor tissues were evaluable in 21 patients (responders, *n* = 6; nonresponders, *n* = 15), baseline plasma EBV-DNA levels were evaluable in 23 patients (responders, *n* = 7; nonresponders, *n* = 16), and baseline T cell, B cell, and NK cell counts and other immune profiling results were evaluable in 23 patients (responders, *n* = 7; nonresponders, *n* = 16).

### Biomarker sample collection

Fresh tumor samples were collected for assessment of CD38 expression from core needle biopsy within 21 days of cycle 1 day 1 (if unavailable, archived formalin-fixed, paraffin-embedded blocks/slides were acceptable). Whole blood samples (~ 12 mL) were collected for assessment of complement proteins and immunophenotyping prior to infusion on day 1 of cycles 1, 2, 3, 6, and 7 and at the end-of-treatment visit. An additional 4 mL of blood was collected monthly and at the end-of-treatment visit for circulating plasma EBV-DNA quantification at the central laboratory as a biomarker of tumor load. Clinically nonevaluable patients were excluded from biomarker analysis.

### Circulating plasma EBV-DNA quantification

Four milliliters of blood was drawn into an EDTA tube and mixed immediately by gentle inversion. Samples were centrifuged (1500–2000 × g) for ≥ 15 min until the cells and plasma were separated, and the plasma layer was tested for EBV-DNA titer in a central laboratory. Plasma EBV-DNA titer was measured via quantitative polymerase chain reaction (PCR) assay with a lower limit of quantitation of 45 IU/mL (tested at Viracor Eurofins Clinical Diagnostics, Summit, MO, USA).

### Immunohistochemistry (IHC) for CD38 detection

CD38 expression on tumor tissue was assessed by IHC centrally using rabbit anti-human CD38 monoclonal antibody (SP149; Cell Marque, Rocklin, CA, USA), according to the previously described protocol [20].

### Complement protein assessment

Six milliliters of blood was collected for complement protein-level assessment (C1q complex, C2, C3, and C4). Serum CIC (circulating immune complexes)-C1q levels were measured by ELISA (MicroVue™ CIC-C1q EIA, QUIDEL), and serum C2 levels were measured by radial immunodiffusion at the laboratory of National Jewish Health (Denver, CO, USA). Serum C3 and C4 levels were measured using NK023.S and NK025.S kits, respectively, on the SPAplus turbidimetric analyzer (The Binding Site, Birmingham, UK).

### Immune cell phenotyping and quantification by flow cytometry

Peripheral blood (PB) was collected in heparinized tubes at baseline, prior to the first infusion, and at specified time points during treatment, and evaluated using flow cytometry within 24 to 48 h of collection in central laboratories. Peripheral blood mononuclear cells (PBMCs) were isolated from 4 mL whole blood by density-gradient centrifugation. Samples were stained with the indicated antibody panels (details in the Supplemental Appendix).

### T cell receptor (TCR) and B cell receptor *(*BCR) clonal sequencing

ImmunoSEQ^®^ assay (Adaptive Biotechnologies, Seattle, WA, USA) was used for TCR and BCR repertoire characterization. Genomic DNA from frozen PBMCs (2 mL blood) was assessed by a multiplex PCR-based method that amplifies rearranged TCR and BCR complementarity-determining region 3 (CDR3) sequences, utilizes capacity of high throughput sequencing, and characterizes tens of thousands of corresponding T cell receptor beta (TCRβ) and BCR immunoglobulin heavy chain (IGH) CDR3 chains accordingly [21, 22]. The multiplex PCRs were composed of forward and reverse primers directly targeting the family of variable (V) genes (forward primers) and joining (J) genes (reverse primers). Each V and J gene primer acted as priming pairs to amplify somatically recombined TCRs or BCRs. Following initial PCR amplification, each amplicon was amplified again with forward and reverse primers containing the universal and adaptor sequences needed for DNA sequencing by Illumina.

### Cytometry by time-of-flight (CyTOF) staining and acquisition

For CyTOF analysis, 2 mL whole blood samples were collected, fixed in Smart Tubes (Smart Tube, Inc., Las Vegas, NV, USA) per manufacturer’s instructions, and stored at – 80 °C until analysis. Samples were thawed by gentle shaking in a 10 °C water bath for 20 min. Red blood cells were initially lysed by the addition of Thaw-Lyse Buffer (Smart Tube, Inc.) followed by BD Pharm Lyse lysing solution (BD Biosciences, Franklin Lakes, NJ, USA), according to the manufacturers’ protocol. Cells were washed with staining buffer and barcoded using Cell-ID 20-Plex Pd Barcode Kit (Fluidigm) then washed and pooled for subsequent staining. Surface Fc receptors were blocked using Human TruStain FcX™ (BioLegend, San Diego, CA, USA) for 15 min at room temperature followed by staining with an antibody cocktail against surface markers for 30 min at room temperature. Samples were washed with staining buffer and prepared for intracellular staining using BD Cytofix/Cytoperm fixation/permeabilization kit (BD Biosciences), according to the manufacturer’s instructions. Samples were stained with a cocktail of intracellular antibodies for 30 min at 4°C, washed, resuspended in phosphate-buffered saline containing 0.05 μM Iridium-DNA Intercalator (Fluidigm, San Francisco, CA, USA) and 1.6% methanol-free formaldehyde solution, and incubated overnight at 4 °C until CyTOF measurements.

All antibodies for CyTOF were either purchased conjugated from Fluidigm or conjugated in-house using Maxpar X8 and Maxpar MCP9 antibody labeling kits (Fluidigm) according to the manufacturer’s recommended protocols. A complete summary of the CyTOF panel used for the analysis is presented in Supplemental Table 1.

### CyTOF data acquisition and processing

On the day of acquisition, samples were washed with staining buffer, followed by cell acquisition solution (CAS; Fluidigm), counted, and resuspended in CAS containing 1:10 diluted EQ™ Four Element Calibration Beads at 0.65 × 10^6^ cells/mL. Samples were acquired on a CyTOF2 system upgraded to Helios specifications with a flow rate of 250 to 350 events/second.

Flow Cytometry Standard (FCS) files were normalized and debarcoded using FCS-processing and debarcoding modules within the CyTOF software. Manual gating of immune populations of interest was performed using the Cytobank platform (Beckman Coulter, Indianapolis, IN, USA), and data were further processed via custom scripts based on the flowCore package. Channel intensities were normalized with calibration beads following data acquisition, and the arcsinh function (cofactor = 5) was used to transform measured intensities for each channel. Samples with > 10,000 live singlet events of lymphocyte/monocyte count were clustered into nodes of similar cellular events using the spanning-tree progression analysis of density-normalized events (SPADE) algorithm using Cytobank software [23, 24]. Clusters were grouped into bubbles corresponding to known populations based on intensity profiles. Quality control was performed using the HilbertSimilarity distance algorithm [25], Earth Mover’s Distance algorithm [26], and Marker Enrichment Modeling [27]. These analyses revealed the absence of technical batch effects and the expected clustering of control samples.

### Statistical analysis

Negative binomial regression was used to model count data from different cell populations and was able to account for over-dispersion present in the population size. For each cell population, a negative binomial generalized linear mixed model was fitted, from which the contrasts of interest were derived. Each univariate mixed model included response to treatment and time point as the main effects, the interaction between the two variables, and a random patient effect. The total number of cells in the sample was used as the offset term in the models, to normalize the count data for clusters and bubbles. For manually gated populations, two models were built using either the total number of cells in the sample or the number of cells in the parent population as an offset term. Tukey’s method was used for multiple comparison correction for the contrasts considered within each population’s model. Additionally, false discovery rate was applied to correct for testing across different cell populations.

### Visualization

Median marker intensity differential testing results were visualized in a SPADE-Treeblend plot by coloring each SPADE tree cluster using a combination of raw *P* values and fold changes computed after changes in marker intensities or population fractions. Numbers (nodes) grayed out in SPADE trees were not included in the analysis due to a restricted parent-child population comparison or the existence of an empty node for 1 patient sample in the respective dataset. FreeViz projections [28] were used to visualize population-level differences between response to treatment and time point; briefly, cells were projected in the context of channels used for the analysis. Channel positions were updated using a supervised algorithm to maximize distance between cells from different categories. Composition shifts were visualized using density plots and interpreted relative to the channel positions after optimization. Fan charts developed by the Bank of England [29] were used to examine individual contributions of each channel and assess homogeneity of response across a given cell population. Briefly, centiles for each marker and condition were calculated, and corresponding values were visualized as stacked area plots color-coordinated to corresponding centiles. Color intensity is greatest at the center of each fan chart (centered on the 50th centile) and decreases symmetrically across the spectrum.

## Results

### Prognostic factors among responders and nonresponders

**Fig. 1:**
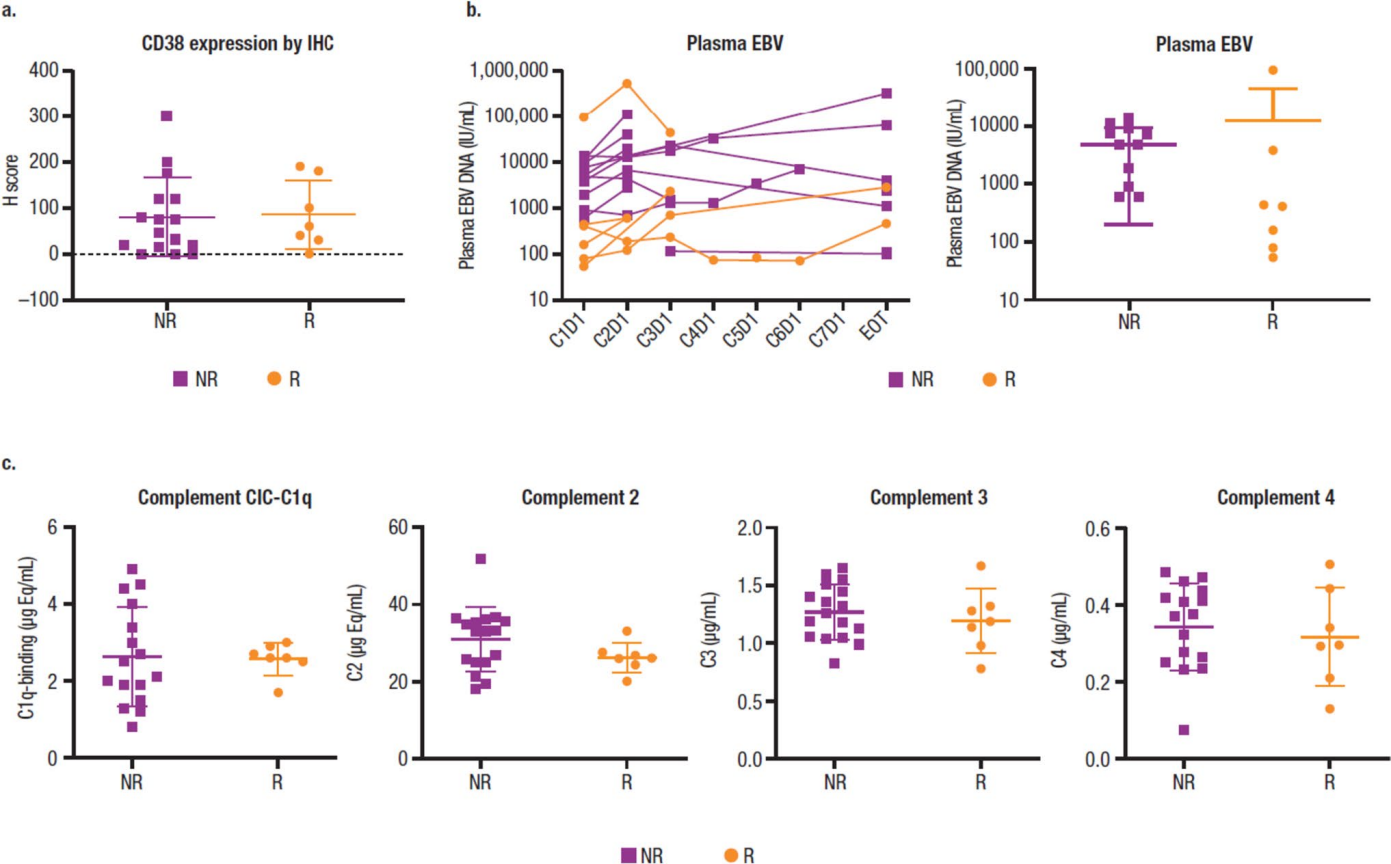
Response to daratumumab is not associated with CD38 expression, baseline EBV-DNA, or serum complement function. **a** CD38 expression by IHC. **b** Plasma EBV levels. **c** Serum complement CIC-C1q, C2, C3, and C4 levels. EBV, Epstein-Barr virus; IHC, immunohistochemical analysis; NR, nonresponder; R, responder; C, cycle; D, day; EOT, end of treatment; CIC, circulating immune complexes

As reported previously [16], baseline tumor CD38 expression and plasma EBV-DNA levels did not clearly correlate with clinical response to daratumumab (Fig. 1a, b). Common prognostic factors for response to standard of care, including β_2_ microglobulin, prognostic index of NK lymphoma, and disease stage, did not predict clinical response to daratumumab (Supplemental Fig. 1). Baseline serum complement protein CIC-C1q, C2, C3, and C4 levels in PB were comparable regardless of clinical response (Fig. 1c). IHC staining of individual main complement inhibitory proteins CD46, CD55, and CD59 in tumor tissue did not show a correlation with clinical response (data not shown).

### Immune repertoire effects and the immunomodulatory role of daratumumab in patients with R/R NKTCL

**Fig. 2:**
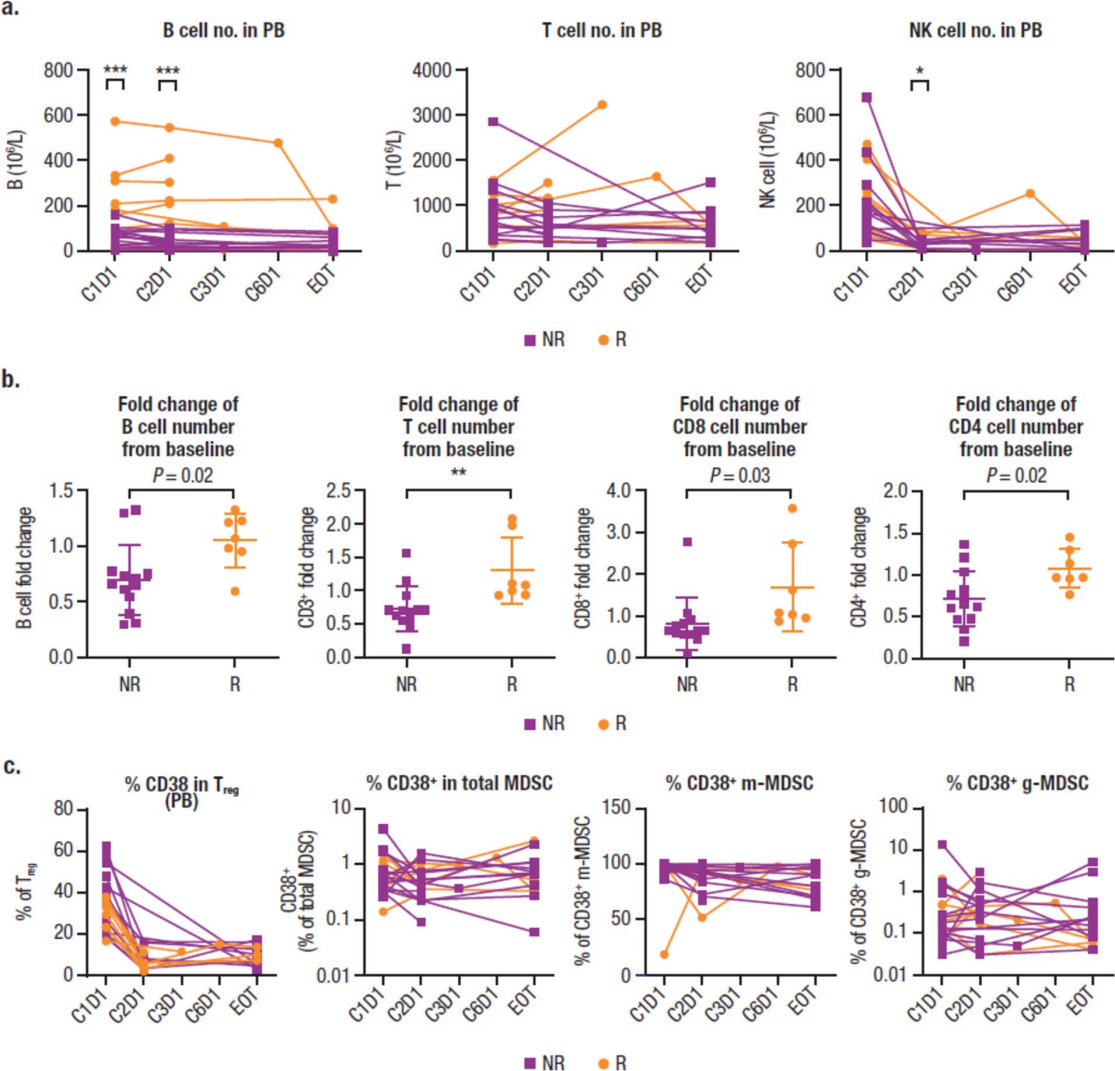
Immune repertoire effects and the immunomodulatory role of daratumumab as measured by MPFC. **a** The number of B, T, and NK cells in PB. **b** Fold changes following daratumumab treatment in the number of B, T, CD8, and CD4 cells from baseline. **c** CD38 percentages in T_reg_, total MDSC, and m-MDSC. MPFC, multiparameter flow cytometry; NK, natural killer; PB, peripheral blood; C, cycle; D, day; EOT, end of treatment; T_reg_, regulatory T cell; MDSC, myeloid-derived suppressor cell; m-MDSC, monocytic myeloid-derived suppressor cell; g-MDSC, granulocyte-like myeloid derived suppressor cell. *P < 0.05; **P < 0.005; ***P < 0.0005

Based on multiparameter flow cytometry (MPFC) data, the baseline number of B cells (CD19^+^) but not T cells (helper/inducer: CD3^+^CD4^+^; cytotoxic: CD3^+^CD8^+^) or NK cells in PB correlated significantly with clinical response to daratumumab (Fig. 2a). A reduction in NK cell (CD3^–^CD16^+^CD56^+^) numbers in PB after treatment was observed in responders and nonresponders (Fig. 2a). B cell counts remained unchanged from baseline in responders after daratumumab treatment, whereas a slight reduction in B cell counts from baseline was observed in nonresponders (Fig. 2b). Baseline B cell count was higher in responders versus nonresponders while baseline T cell and NK cell counts were similar between responders and nonresponders (Supplemental Fig. 2). Consistent with observations in MM patients [9], an increase of T cell numbers (both CD8^+^ and CD4^+^) was seen post-treatment in responders (Fig. 2b), whereas a decrease of T cell numbers was observed post-treatment in nonresponders; in both responders and nonresponders, no significant difference was observed in the CD4/CD8 ratio, as measured by flow cytometry. Although the absolute number of regulatory T cells (T_regs_; CD3^+^CD4^+^CD8^–^CD25^+^CD127^dim^) and total myeloid-derived suppressor cells (MDSCs; HLADRloLin^−^CD33^+^CD11b^+^) did not significantly change after daratumumab treatment (data not shown), the percentage of CD38^+^ T_regs_ and CD38^+^ monocytic MDSCs (m-MDSCs) was reduced to a similar extent in both responders and nonresponders (Fig. 2c).

**Fig. 3:**
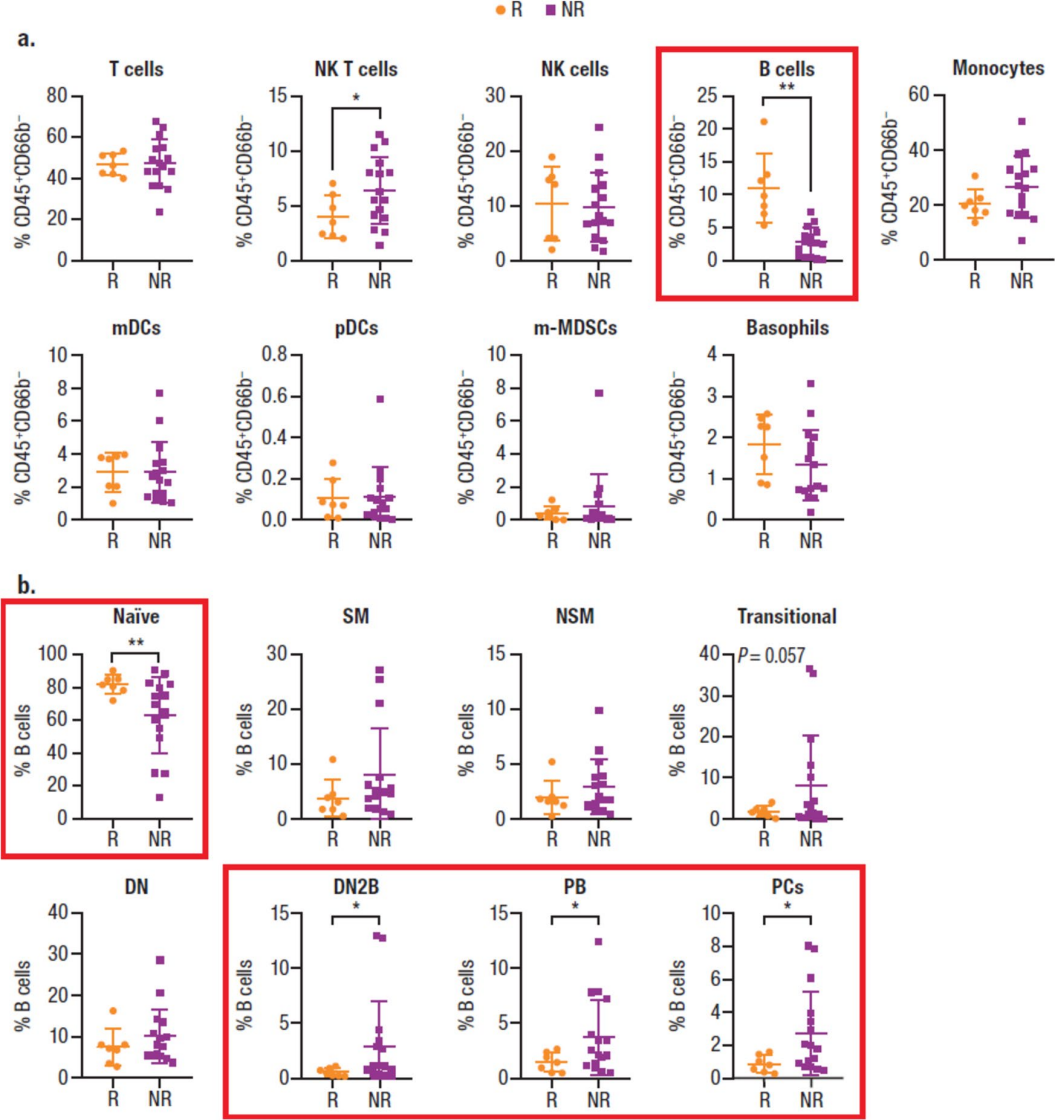
Immune repertoire effects and the immunomodulatory role of daratumumab as measured by CyTOF. **a** Baseline cell percentages. **b** B cell subtype percentages by response (*P* values were calculated with the unpaired *t* test with Welch’s correction). R/R NKTCL, relapsed or refractory natural killer/T cell lymphoma; CyTOF, cytometry by time-of-flight; R, responder; NR, nonresponder; NK, natural killer; mDC, myeloid dendritic cell; pDC, plasmacytoid dendritic cell; m-MDSC, monocytic myeloid-derived suppressor cell; SM, switched memory; NSM, nonswitched memory; DN, double negative; DN2, double negative 2; PB, plasmablast; PC, plasma cell. Graphs with particular significance are denoted with red boxes. *P < 0.05; **P < 0.01

**Fig. 4:**
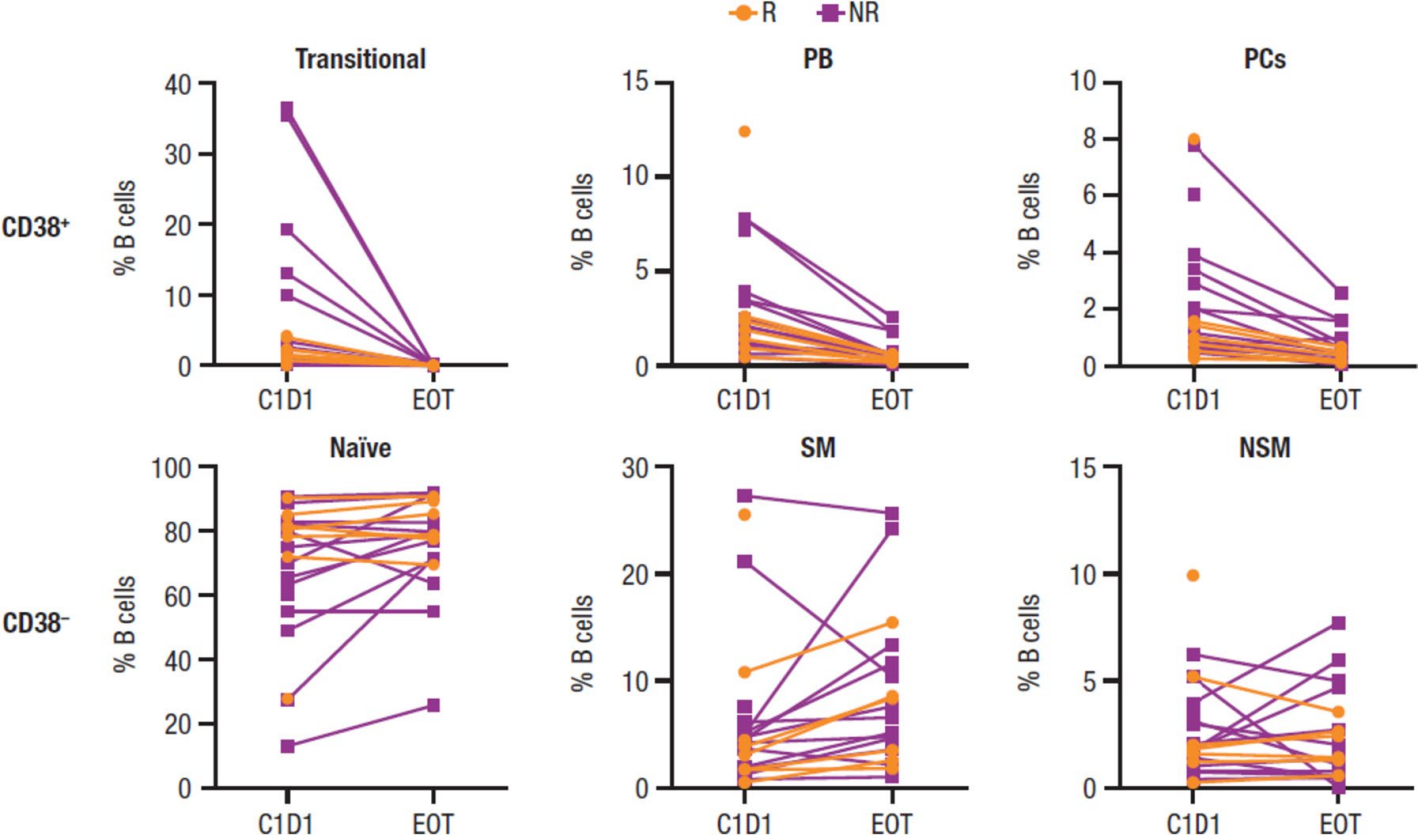
Change in the percentage of CD38^+^ and CD38^–^ B cell subtypes after daratumumab treatment. R, responder; NR, nonresponder; PB, plasmablast; PC, plasma cell; C, cycle; D, day; EOT, end of treatment; SM, switched memory; NSM, nonswitched memory

CyTOF analyses, which allowed a deep dive into cellular components of the NKTCL-patient peripheral immune response, confirmed a higher percentage of B cells at baseline in responders versus nonresponders (Fig. 3a), consistent with findings obtained by MPFC. High percentages of baseline naïve B cells, most of which are CD38^–^, correlated with clinical response (Fig. 3b). Additionally, low percentages of double-negative 2 B cells (DN2; a novel subset of CD27^–^IgD-B cells that are CD38^–^CD24^–^ but express CD11c), plasmablasts, and plasma cells, most of which are CD38^+^, also correlated with clinical response. CD11c^+^ DN2 B cells, which have recently been implicated in autoimmune diseases, represent a memory B cell subset with a capacity to differentiate into antibody-producing plasma cells [30–32]. Additionally, previous data indicate that B cells can differentiate into plasma cells in the vaccine setting for daratumumab-treated MM patients [33]. Thus, maintenance of CD38^–^ naïve B cells and depletion of the small percentage of plasmablasts and plasma cell B subtypes after daratumumab treatment are expected to alter BCR immune repertoire without changing overall B cell percentages (Fig. 4).

**Fig. 5:**
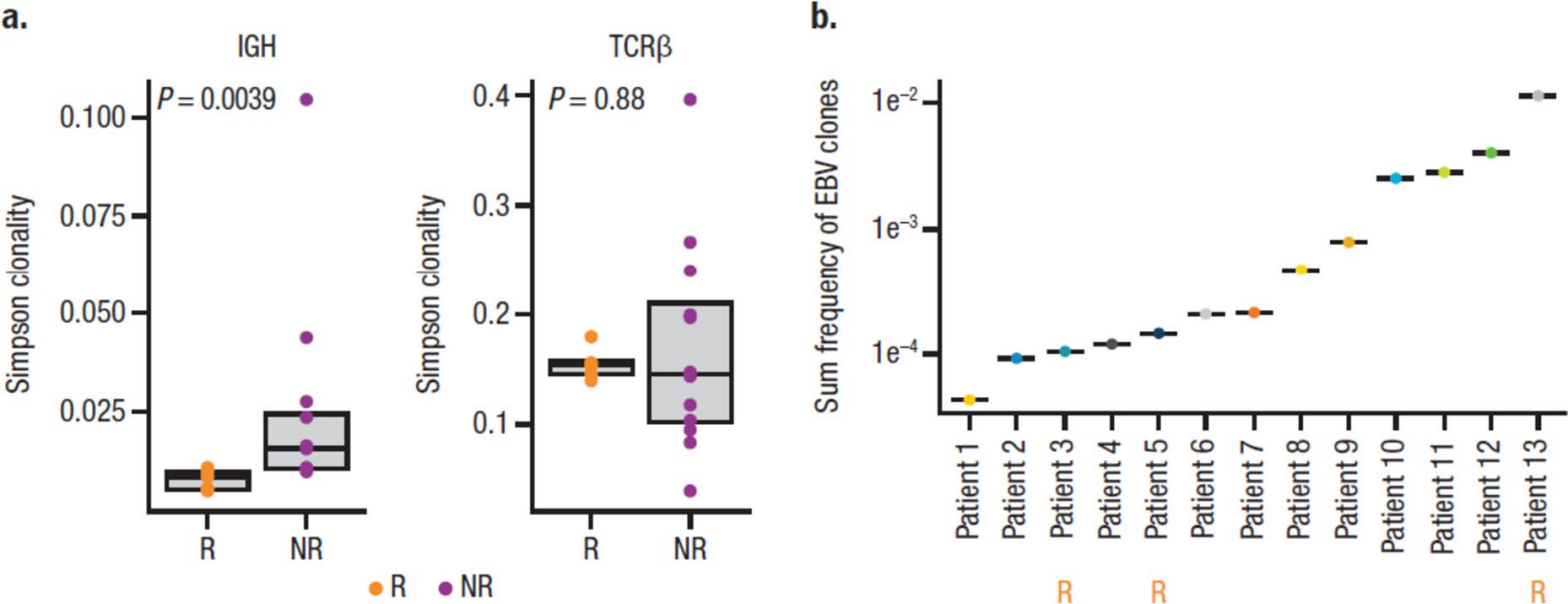
B cell and EBV clonality in patients with R/R NKTCL. **a** Baseline B cell clonality by response (*P* values were calculated with the Wilcoxon test). **b** Frequency of EBV-related clones in each evaluable patient. EBV, Epstein-Barr virus; R/R NKTCL, relapsed or refractory natural killer/T cell lymphoma; IgH, immunoglobulin heavy chain; TCRβ, T cell receptor beta; R, responder; NR, nonresponder

**Fig. 6:**
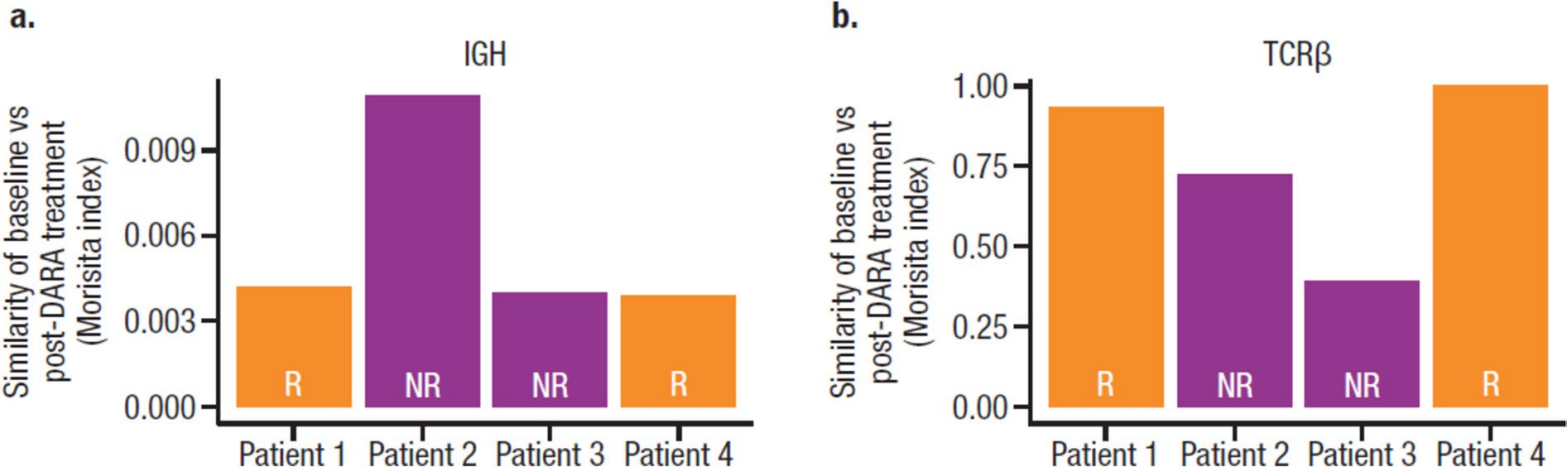
Clonality similarity between baseline versus post-daratumumab treatment. **a** B cell clones. **b** T cell clones. DARA, daratumumab; IgH, immunoglobulin heavy chain; TCRβ, T cell receptor beta; R, responder; NR, nonresponder. Morisita index: a similarity metric that ranges from 0 to 1, where a value of 1 indicates identical repertoires and a value of 0 indicates no sequences are shared between 2 samples

To determine whether daratumumab treatment altered the clonal identity of B cells, a representative subgroup of 13 patients, including 2 responders and 2 nonresponders with post-treatment PBMC samples, underwent BCR and TCR sequencing. Since EBV-encoded RNA in situ hybridization positivity is a diagnostic criteria of NKTCL [34], we hypothesized that responders could have large numbers of baseline EBV-targeting B clones directed against the underlying EBV infection and would have a less diverse BCR repertoire. Sequencing of BCR IGH and TCRβ locus revealed lower BCR clonality in responders (measured by Simpson clonality), whereas TCR clonality was not affected (Fig. 5a). Therefore, it is unlikely responses can be fully explained by pre-existing EBV-related B cell clones. Furthermore, we asked whether EBV-related B cells could be detected in patients. Subsequent annotation of baseline clonal sequence and matching to known EBV-associated sequence indicated that all patients had ≥ 1 EBV-related clone; the patient with the highest sum frequency of EBV-related clones at baseline was a responder (Fig. 5b). However, there were no clear relationships between pre-existing or treatment-emergent EBV-related B or T cell clones with clinical response. Furthermore, daratumumab treatment did not alter repertoire diversity (measured by down sample rearrangements) of B or T cell lineages based on response (data not shown). However, in the limited samples with available longitudinal clonality data, only 0.4 to 1% of the BCR repertoire was similar between baseline and post-treatment samples, while there was 40 to 100% similarity between TCR repertoire, suggesting the entire BCR repertoire was restructured following daratumumab treatment (Fig. 6).

**Fig. 7:**
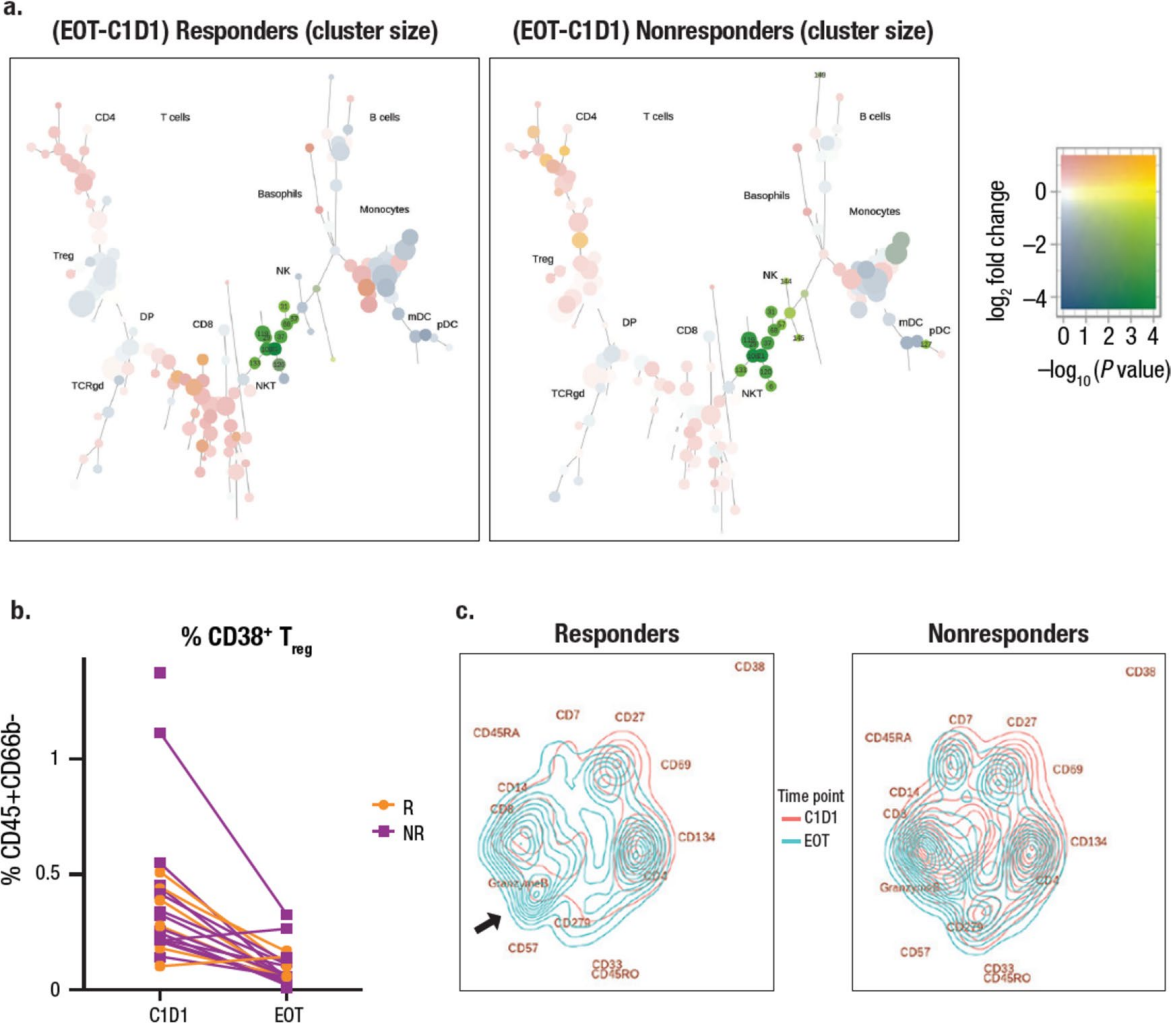
Visualizing daratumumab-mediated changes in immune cells. **a** CyTOF analysis of main cell population changes after daratumumab treatment visualized by clustering. **b** CyTOF analysis of population changes in CD38^+^ NK T cells with daratumumab treatment visualized by a line graph. **c** FreeViz visualization of functional changes in T cells after daratumumab treatment. CyTOF, cytometry by time-of-flight; EOT, end of treatment; C, cycle; D, day; T_reg_, regulatory T cell; DP, double positive; TCRgd, T cell receptor gamma delta; mDC, myeloid dendritic cell; pDC, plasmacytoid dendritic cell; R, responder; NR, nonresponder

In addition to B cell subtypes and their correlation to clinical response, CyTOF analysis demonstrated a reduction of CD38^+^ NK cells, and NK T cells with daratumumab treatment, as well as a reduction in MDSCs and T_regs_ within the CD38^+^ fraction (Fig. 7a, b). We used Treeblend plots to visualize significant changes in population size in different conditions. The reduction in T_regs_ was more substantial in responders than in nonresponders (compare left and right panels), and there was an increase in CD8^+^ T cells in responders but not in nonresponders. Peripheral NK cells were diminished upon daratumumab treatment in both responders and nonresponders. Persisting NK cells in patients with R/R NKTCL express cell surface markers characteristic of an immature phenotype and dysfunctionality (Supplemental Fig. 3). The 4 main nodes identified were immature CD57^–^HLA^–^DR^+^CD56^bright^ NK cells. Although node 144 expressed functional marker CD16 and all nodes expressed granzyme B and perforin, low CD137 expression was observed in all nodes, indicating that the persisting NK cell functions are likely compromised. To further explore the effect of daratumumab on T cells, we trained a FreeViz map [28] to visualize differences in cell composition between time points and between responders and nonresponders. After training, cells were visualized as density plots, whereas cell surface markers most relevant to the separation between time points and responders and nonresponders are far away from the center of the projection. Daratumumab treatment clearly shifted the composition away from CD38 in responders and nonresponders, suggesting reduced CD38 expression in all populations post-treatment. In responders, composition shifted towards granzyme B^+^ CD57^+^ terminally differentiated CD8^+^ T cells (left panel, arrow) compared to nonresponders (right panel, Fig. 7c). Higher density in the CD8^+^ population compared with the CD4^+^ population was observed in responders versus nonresponders, in whom density of CD8^+^ was much less, suggesting a unique increase of CD8^+^ in responders (Fig. 7c). The same trend towards a decrease in the CD4/CD8 ratio from baseline in responders but not nonresponders was also observed by direct gating of CyTOF populations (Supplemental Fig. 4). Total frequency of T_regs_ and MDSCs did not change despite diminished CD38 expression in both responders and nonresponders (Supplemental Fig. 5), suggesting that daratumumab did not reverse the immune suppression mediated by T_regs_ or MDSCs.

## Discussion

Extranodal NKTCL, nasal type, is a rare, fast-growing (high-grade) non-Hodgkin lymphoma. It is more common in people from Asia, Central America, and South America, but it is very rare in the western world. It can start in T cells, but it develops most often in NK cells. In general, neoplastic NK cells exhibit a larger proportion and homogeneous population with a greater intensity of CD56 expression and forward scatter level compared with reactive NK cells, as well as a homogeneous positive CD38 phenotype [35].

This investigation of the NKT2001 study, to our knowledge, is the first in-depth B cell subtype analysis and first observation of the BCR repertoire in daratumumab-treated patients with R/R NKTCL. As demonstrated by MPFC, treatment with daratumumab led to a reduction in the absolute numbers of NK cells and the percentage of CD38^+^ T_regs_ and CD38^+^ m-MDSCs in responders and nonresponders, as well as an increase in the number of CD4^+^ and CD8^+^ T cells with a concomitant decrease of CD4/CD8 ratio in responders but not nonresponders. A lower reduction of total B cell counts observed in responders versus nonresponders suggests that the differential response may be correlated with specific B cell subtypes or additional cell types unable to be measured by MPFC. Analysis by CyTOF supported the MPFC findings, and further indicated a specific reduction in the number of plasma cells and plasmablasts, but not in naïve B cells with daratumumab treatment. Data show that high percentages of baseline naïve B cells and low percentages of DN2 B cells, plasmablasts, and plasma cells are correlated with clinical response. However, unclear relationships between EBV-related B or T cell clones (pre-existing or treatment-emergent) with clinical response may have been confounded by MHC restriction of EBV antigens in the analyses. The high number of baseline B cells did not correlate directly with greater EBV-specific immune clones; however, responders presented with the highest number of baseline EBV-related clones. Daratumumab treatment induced a shift in the composition of the T cell compartment in responders, increasing the fraction of terminally differentiated CD8^+^ T cells. Overall, daratumumab did not significantly alter diversity of B or T cells based on response; however, daratumumab did restructure B cell clone repertoire without impacting T cell clonal identity.

Immune profiling of patients with R/R NKTCL demonstrated maintenance of CD38^–^ naïve B cells and depletion of plasmablasts and plasma cell B subtypes following daratumumab treatment. The findings from NKT2001 indicate that CD38 activity contributes to the pathogenic roles of autoreactive B cell populations and potentially modulates the diverse immune cell types in the autoimmune setting. This phenomenon has been observed in pre-clinical data using the anti-CD38 antibody TAK-079 in a cynomolgus primate model of collagen-induced arthritis [36]. Korver et al. showed that histomorphometric and radiological analyses revealed significantly less joint damage in animals treated prophylactically and therapeutically with TAK-079, and this benefit relates to the depletion of CD38-expressing leukocytes. Daratumumab treatment was also found to reduce the frequency of normal plasma cells in bone marrow samples from patients with relapsed or refractory MM, as observed through reduced levels of polyclonal IgA, IgE, and IgM [33].

In MM, the on-tumor and immunomodulatory mechanism of action of daratumumab has been well described [5–12]. In NKTCL, further investigation is needed to determine if the immunomodulatory activities of daratumumab confer clinical benefit among patients with CD38-negative tumors. The finding that one patient in NKT2001 with CD38-negative tumors responded to treatment suggests that the immunomodulatory activities of daratumumab may provide clinical benefit among patients whose tumors do not express CD38; however, the small sample size limits this interpretation. Future studies are warranted to examine the immunomodulatory activities of daratumumab and their ability to improve clinical outcomes in patients with CD38-negative tumors.

Finally, other studies have uncovered the role of CD38 in chronic autoimmune diseases, such as inflammatory bowel disease and multiple sclerosis, through various mechanisms [37], and studies evaluating the efficacy of daratumumab in these patient populations may be warranted. In addition, studies have investigated the potential of daratumumab to treat common vasoactive-mediated allergic reactions and post-transplant autoimmune hemolytic anemia [38, 39].

## Conclusions

In summary, although daratumumab induces on-tumor activity, through several CD38 immune-mediated and CD38-modulating actions, and induces immunomodulatory effects in patients with MM, its effects on CD38-expressing R/R NKTCL tumor cells as observed in the NKT2001 study were suboptimal. In this study, the changes observed in the immune profile of patients with R/R NKTCL suggest that modulation of the immune environment, including differences in B and T cell populations among responders and nonresponders, is crucial for daratumumab-mediated anti-tumor activities in this patient population. More broadly, the ability of daratumumab to restructure the B cell clone repertoire while maintaining T cell clonal identity, coupled with its ability to mediate immune responses, may support potential clinical benefits of daratumumab in the autoimmune disease setting.

### Supplementary information

ESM 1 (DOCX 755 KB)

## Data Availability

The data sharing policy of Janssen Pharmaceutical Companies of Johnson & Johnson is available at https://www.janssen.com/clinical-trials/transparency. As noted on this site, requests for access to the study data can be submitted through Yale Open Data Access (YODA) Project site at http://yoda.yale.edu.
