# Supplementary material for: Immune profiling of patients with extranodal natural killer/T cell lymphoma treated with daratumumab
Source: Ann Hematol. 2024 Jan 18;103(6):1989–2001. doi: 10.1007/s00277-023-05603-w (PMC11090967; doi:10.1007/s00277-023-05603-w)
Supplement: Supplementary file 1 — (DOCX 755 KB) [file 277_2023_5603_MOESM1_ESM.docx]

# Supplemental Appendix for Qing et al. Immune profiling of patients with extranodal natural killer/T-cell lymphoma treated with daratumumab

**Methods**

*Immune Cell Phenotyping and Quantification by Flow Cytometry*

T, B, and natural killer cell (TBNK) standard panel consisted of: FITCa-CD3 (SK7; BD Biosciences), PEa-CD16 (B73.1; BD Biosciences), PEa-CD56 (NCAM16.2; BD Biosciences), PE-Cy^™^7a-CD4 (SK3; BD Biosciences), PerCP-Cy^™^5.5a-CD45 (2D1; BD Biosciences), APCa-CD19 (SJ25C1; BD Biosciences), APC-Cy^™^7a-CD8 (SK1; BD Biosciences); myeloid-derived suppressor cell (MDSC) panel consisted of: FITCa-CD15 (W6D3; BD Pharmingen), PEa-CD33 (WM53; BioLegend), PerCPa-CD16 (3G8; BioLegend), AF700a-CD3 (UCHT1; BioLegend), AF700a-CD56 (B159; BD Pharmingen), AF700a-CD19 (HIB19; BD Pharmingen), AF700a-CD20 (2H7; BD Pharmingen), V450a-CD14 (MψP9; BD Biosciences), BV510a-CD11b (ICRF44; BD Biosciences), and BV605a-HLA-DR (L243; BioLegend); regulatory T cells (T_reg_) panel consisted of: FITCa-CD25 (2A3; BD Biosciences), PEa-CD137 (4B4-1; BioLegend), AF700a-CD3 (OKT3; BioLegend), BV421a-CD127 (HIL-7R-M21; BD Pharmingen), BV510a- CD8 (SK1, BD Horizon), and BV605a- CD4 (RPA-T4; BD Horizon). In addition, all samples were stained with AF647a-CD38 (HuMax-003; Janssen) in MDSC and T_reg_ panels.

# Supplemental Table 1 CyTOF Panel of Antibodies

| **Metal label** | **Antigen** | **Clone** | **Source** | **Catalogue #** | **Cocktail** | **SPADE clustering** |
| --- | --- | --- | --- | --- | --- | --- |
| 89Y | CD45 | HI30 | Fluidigm | 3089003B | Surface | NO |
| 111Cd | CD7 | CD7-6B7 | Biolegend | 343111 | Surface | YES |
| 112Cd | TCRvd2 | B6 | Biolegend | 331402 | Surface | YES |
| 113In | CD66b | 913542 | R&D Systems | MAB4246 | Surface | NO |
| 114Cd | CD95 | DX2 | Biolegend | 305631 | Surface | NO |
| 115In | CD57 | NK-1 | BD Biosciences | 555618 | Surface | YES |
| 116Cd | CD11c | Bu15 | Biolegend | 337221 | Surface | YES |
| 141Pr | IgD | IA6-1 | Biolegend | 348235 | Surface | YES |
| 142Nd | CD19 | HIB19 | Biolegend | 302247 | Surface | YES |
| 143Nd | CD279 | EH12.2H7 | Biolegend | 329941 | Surface | NO |
| 144Nd | CD11b | ICRF44 | Biolegend | 301337 | Surface | YES |
| 145Nd | Granzyme B | GB11 | Novus Biologicals | NBP1-50071 | Intracellular | NO |
| 146Nd | CD8 | RPA-T8 | Biolegend | 301053 | Surface | YES |
| 147Sm | CD1d | 51.1 | Biolegend | 350302 | Surface | NO |
| 148Nd | CD152 | L3D10 | Biolegend | 349902 | Intracellular | NO |
| 149Sm | CD134 | ACT35 | Biolegend | 350015 | Surface | NO |
| 150Nd | CD138 | MI15 | BD Biosciences | 551902 | Surface | NO |
| 151Eu | CD14 | M5E2 | Biolegend | 301843 | Surface | YES |
| 152Sm | Perforin | dG9 | Biolegend | 308102 | Intracellular | NO |
| 153Eu | CD45RA | HI100 | Biolegend | 304143 | Surface | YES |
| 154Sm | CD137 | 4B4-1 | Biolegend | 309802 | Surface | NO |
| 155Gd | CD27 | L128 | Fluidigm | 3155001B | Surface | YES |
| 156Gd | CD45RO | UCHL1 | Biolegend | 304239 | Surface | YES |
| 157Gd | Cleaved Caspase-3 | C92-605 | BD Biosciences | custom | Intracellular | NO |
| 158Gd | CD33 | WM53 | Biolegend | 303419 | Surface | YES |
| 159Tb | CD244 | C1.7 | Biolegend | 329502 | Surface | NO |
| 160Gd | CD69 | FN50 | Biolegend | 310939 | Surface | NO |
| 161Dy | TIM3 | 344823 | R&D Systems | MAB2365 | Surface | NO |
| 162Dy | CD40 | 5C3 | Biolegend | 334325 | Surface | NO |
| 163Dy | CD56 | R19-760 | Biolegend | 563237 | Surface | YES |
| 164Dy | CD15 | W6D3 | Fluidigm | 3164001B | Surface | NO |
| 165Ho | CD38 | HuMax | Janssen | custom | Surface | NO |
| 166Er | CD127 | eBioRDR5 | ThermoFisher | 16-1278-82 | Surface | YES |
| 167Er | CD24 | ML5 | Biolegend | 311127 | Surface | NO |
| 168Er | VISTA | 730804 | R&D Systems | MAB71261 | Surface | NO |
| 169Tm | CD25 | M-A251 | BD Biosciences | 555429 | Surface | YES |
| 170Er | CD3 | UCHT1 | Biolegend | 300443 | Surface | YES |
| 171Yb | CD20 | 2H7 | Biolegend | 302343 | Surface | YES |
| 172Yb | CD123 | 9F5 | BD Biosciences | 555642 | Surface | YES |
| 173Yb | CD314 | 1D11 | Biolegend | 320814 | Surface | NO |
| 174Yb | HLA-DR | L243 | Biolegend | 307651 | Surface | YES |
| 175Lu | CD274 | 29E.2A3 | Fluidigm | 3175017B | Surface | NO |
| 176Yb | CD4 | RPA-T4 | Biolegend | 300541 | Surface | YES |
| 194Pt | CD28 | CD28.2 | Biolegend | 302937 | Surface | NO |
| 198Pt | CD44 | IM7 | Biolegend | 103051 | Surface | NO |
| 209Bi | CD16 | 3G8 | Fluidigm | 3209002B | Surface | YES |

# Supplemental Fig. 1 β_2_ microglobulin, PINK, and disease stage versus clinical response


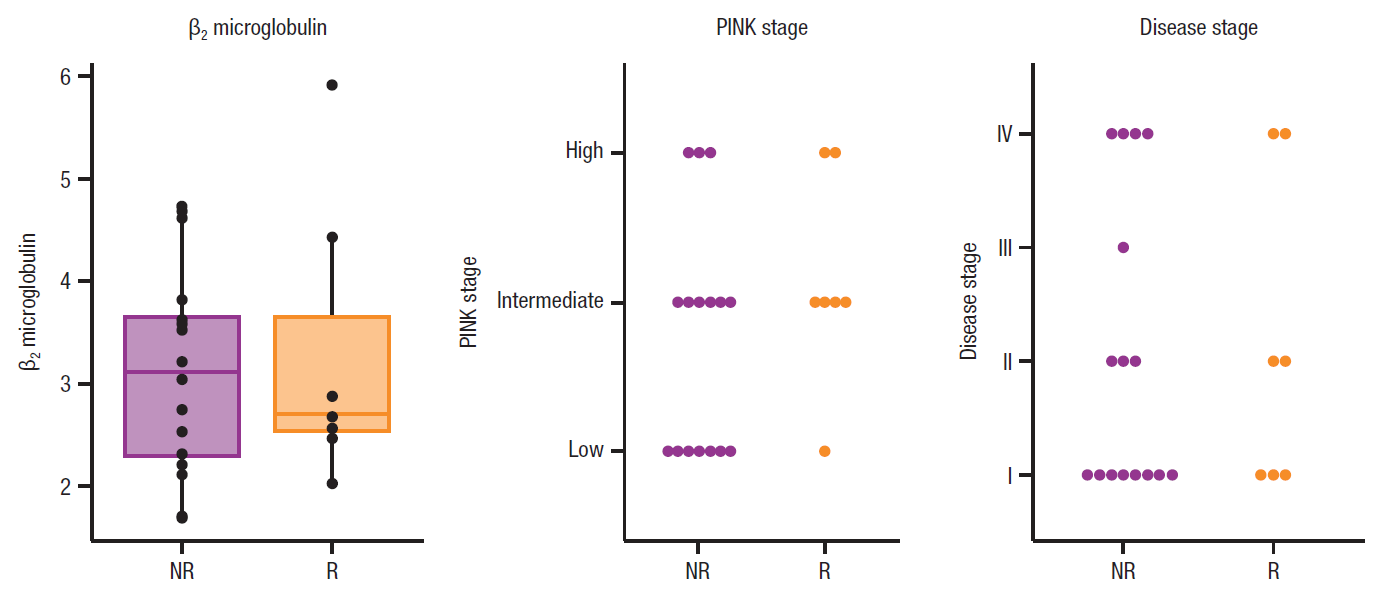


Common prognostic factors for patient response to standard of care treatment did not predict clinical response to daratumumab.

PINK, prognostic index of natural killer lymphoma; NR, nonresponder; R, responder.

# Supplemental Fig. 2 Baseline B, T, and NK cell levels in responders versus nonresponders


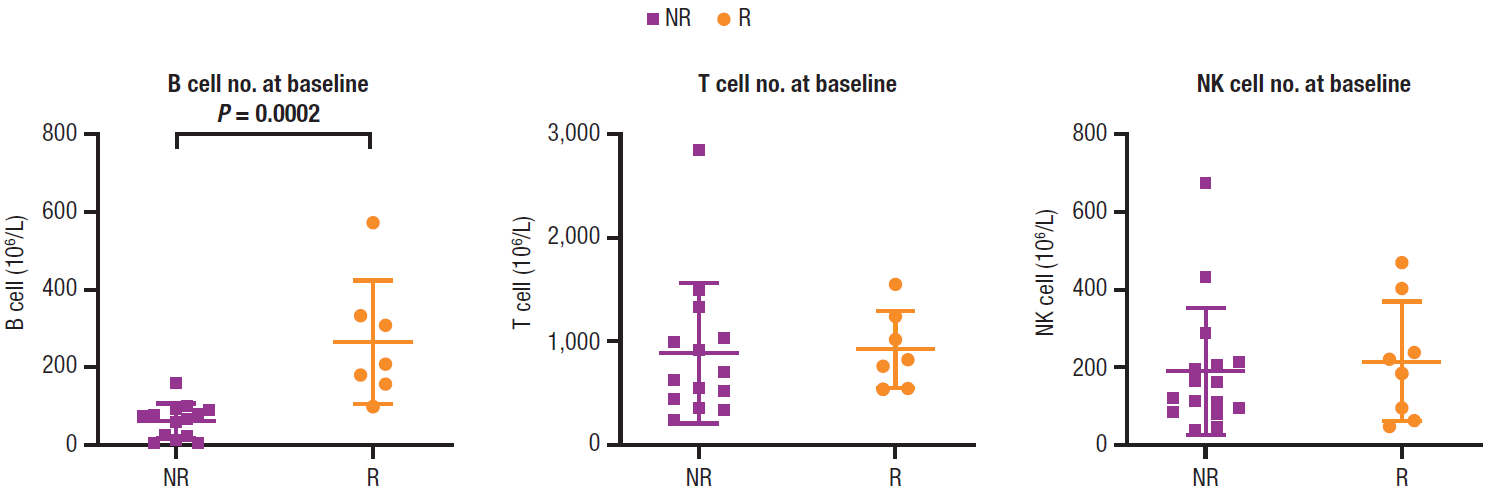


The difference observed in baseline B, T, and NK cell levels by response.

NK, natural killer; NR, nonresponder; R, responder.

**Supplemental Fig. 3** **Persisting NK cells identified by CyTOF**


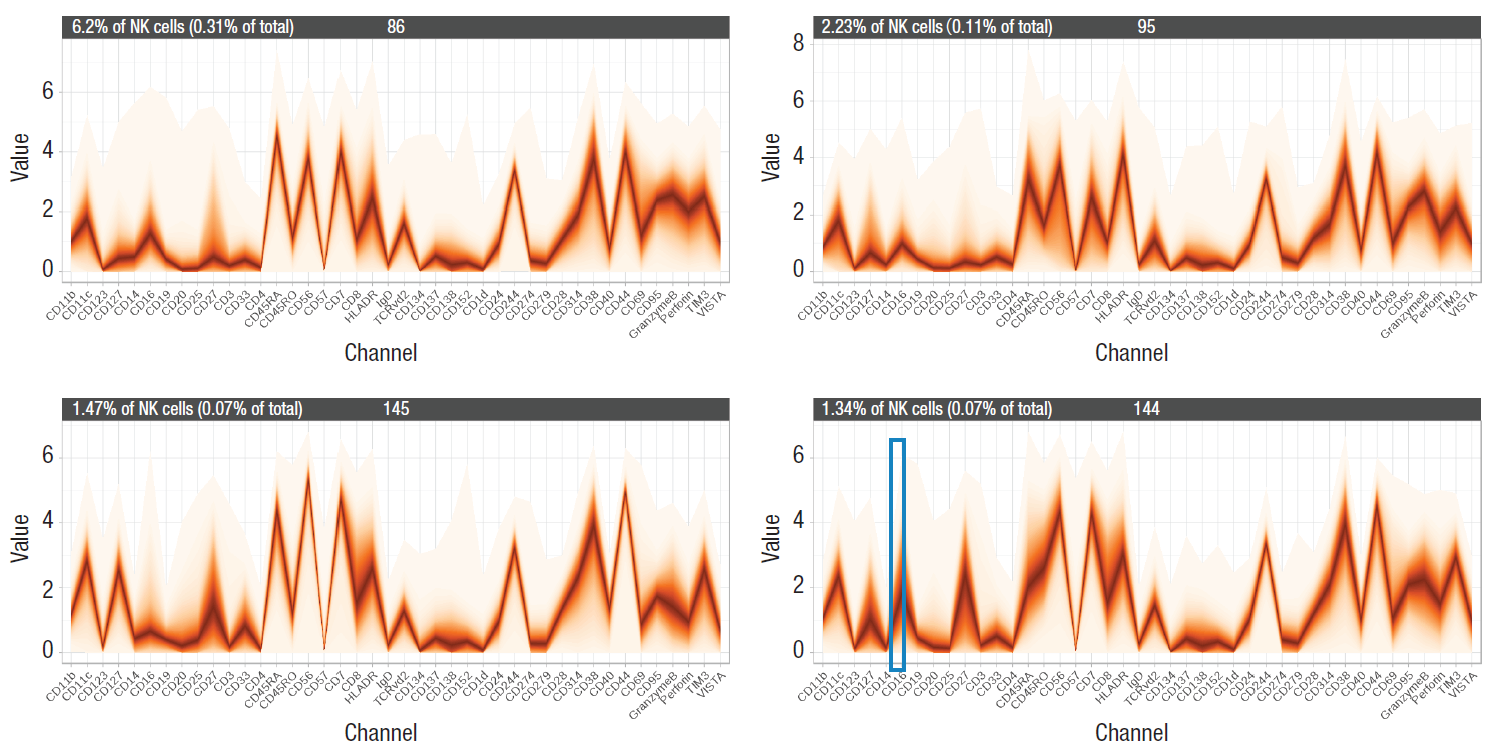


Fan chart of persisting NK cells in NKTCL patients – the 4 main nodes identified are immature CD57^−^HLA^−^DR^+^CD56^bright^ NK cells. The percentage of NK cells represents the percentage of cells in the cluster relative to the NK cell bubble; percentage of total represents the total number of live cells in the experiment.

Area of particular significance is denoted with the blue box.

NK, natural killer; CyTOF, cytometry by time-of-flight; NKTCL, natural killer/T cell lymphoma.

# Supplemental Fig. 4 Difference in CD4/CD8 ratio in responders versus nonresponders


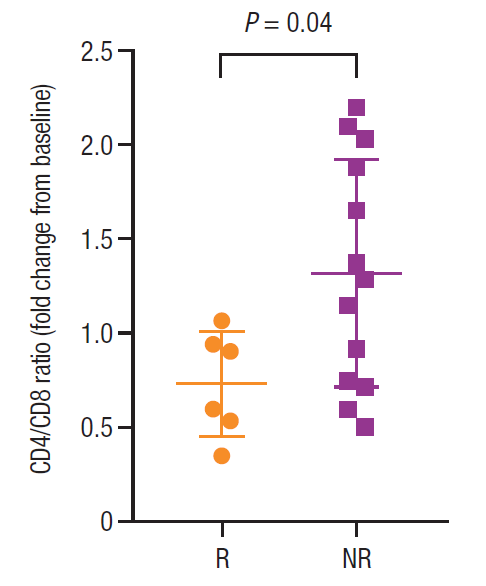


The difference observed in CD4/CD8 ratios was observed by direct gating of CyTOF populations.

R, responder; NR, nonresponder; CyTOF, cytometry by time-of-flight.

*P* value was calculated with the unpaired *t* test.

# Supplemental Fig. 5 Total frequency of a) T_regs_ and b) MDSCs


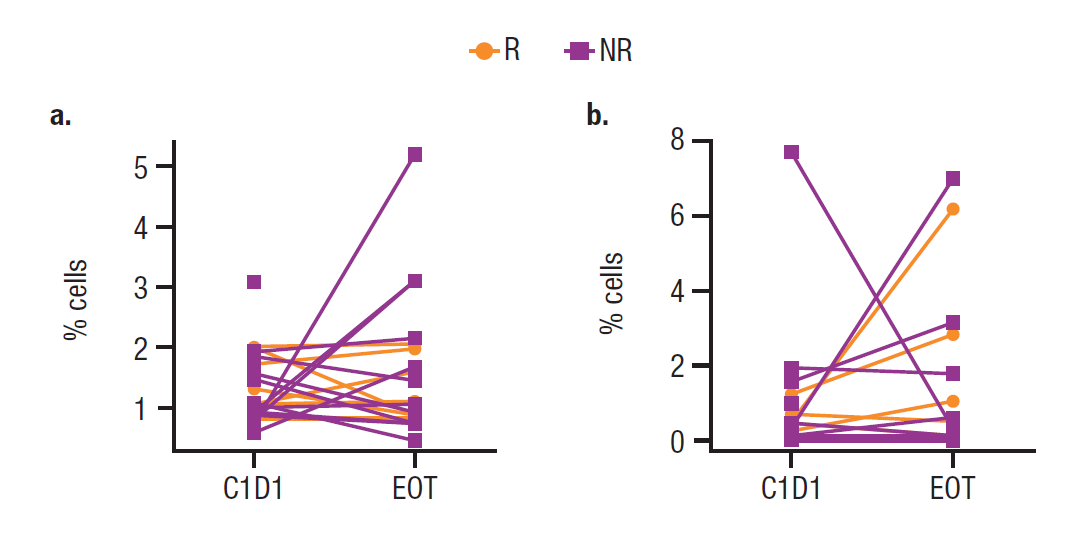


Reduced CD38 expression did not result in a change in the total frequency of T_regs_ and MDSCs.

T_regs_, regulatory T cells; MDSC, myeloid-derived suppressor cell; R, responder; NR, nonresponder; C1D1, Cycle 1 Day 1; EOT, end of treatment.
